# Efficacy of botulinum toxin A combined with extracorporeal shockwave therapy in post-stroke spasticity: a systematic review

**DOI:** 10.3389/fneur.2024.1342545

**Published:** 2024-03-15

**Authors:** Ya-nan Du, Yang Li, Ting-yu Zhang, Nan Jiang, Ying Wei, Shi-huan Cheng, He Li, Hao-yang Duan

**Affiliations:** ^1^Department of Rehabilitation, School of Nursing, Jilin University, Changchun, China; ^2^Department of radiology, First Hospital of jilin University, Changchun, China; ^3^Department of Rehabilitation Medicine, First Hospital of Jilin University, Changchun, China

**Keywords:** botulinum toxin A, extracorporeal shockwave therapy, stroke, spasticity, combination therapy

## Abstract

**Objectives:**

In recent years, there has been an increase in the number of randomized clinical trials of BTX-A combined with ESWT for the treatment of post-stroke spasticity. This has made it possible to observe the benefits of combination therapy in clinical practice. Therefore, this paper reviews the effectiveness of BTX-A in combination with ESWT for the treatment of post-stroke spasticity.

**Methods:**

By October 2023, a systematic review was conducted in the databases PubMed, Cochrane, Embase, Medline, Web of Science, China National Knowledge Infrastructure, Wan Fang Database, China Biology Medicine disc and China Science and Technology Journal Database were systematically searched. We included randomized controlled trials that reported outcome metrics such as MAS, FMA, and MBI score. Studies were excluded if MAS was not reported. The quality of the included studies was assessed by the Cochrane Collaboration’s tool for assessing risk of bias, and the AMSTAR quality rating scale was selected for self-assessment.

**Results:**

A total of 70 articles were included in the initial search, and six were ultimately included. The results of the included studies showed that the combination therapy was effective in reducing MAS scores and improving FMA and MBI scores in patients with spasticity compared to the control group. Combination therapy has also been shown to improve joint mobility and reduce pain in spastic limbs.

**Conclusion:**

Cumulative evidence from clinical randomized controlled trial studies suggests that the combination therapy is effective in reducing lower limb spasticity and improving mobility after stroke. However, more clinical trials are still needed to corroborate the evidence regarding the efficacy of BTX-A combined with shockwave therapy.

**Systematic Review Registration:**

The system review can be searched in the PROSPERO database (CRD42023476654).

## 1 Introduction

The incidence of stroke is increasing (1, 2), and most patients are left with limb hemiparesis disorders, of which muscle spasms are more common (3, 4). Spasticity is a movement disorder characterized by a velocity-dependent increase in muscle tone due to a hyperactive detachment reflex, often accompanied by a hyperactive tendon reflex, and is a form of Upper motor neuron syndrome (UMN) (5). The spasticity that develops after stroke can limit the use of the affected limb, causing pain, contractures or falls and impairing gait (6). However, long-term hypertonia and spasticity can lead to permanent joint contracture deformity, hindering the functional rehabilitation of paralyzed limbs, and is one of the major causes of disability (7, 8). Post-stroke dyskinesia has a significant impact on motor function, balance, gait and ability to perform activities of daily living, and usually, patients can improve with intensive exercise training (9). However, the first issue to deal with in achieving this is the effect of spasticity (muscle stiffness) on muscle tone and flexibility (10). Spasticity can interfere with or stop exercise prescription (11). In stroke patients, the burden of spasticity on patients, caregivers and society is enormous. Healthcare costs associated with stroke patients with spasticity are four times higher compared to those without spasticity (12).

Traditional treatments for spasticity include Botulinum Toxin A (BTX-A) (13, 14), phenol (15), baclofen (16, 17), surgical correction (18), Extracorporeal Shockwave Therapy (ESWT) (19), and rehabilitation (20–23). Botulinum toxin A (BTX-A) is a neurotoxin whose primary site of action is the nerve endings and cranial nuclei. It reduces muscle hyperactivity by inhibiting the release of acetylcholine at the neuromuscular junction (it cleaves the 25 kDa synaptosome-associated protein, which is required for vesicle docking and therefore for neurotransmitter release) (24). Thus, BTX-A injections can reduce spasticity by temporarily paralyzing muscle activity and reducing muscle hypertonia (25, 26). At present, BTX-A is a first-line agent for the treatment of focal and multifocal spasticity (27). Much of the literature has demonstrated that BTX-A injections can treat muscle imbalances, decrease muscle tone, and improve muscle function (27). However, studies have also proposed that BTX-A and placebo have similar effects in reducing spasticity (28). And the maximum efficacy of BTX-A in PSS was seen at about 3–4 weeks, and the clinically significant effect of BTX-A was maintained for about 4–8 weeks, after which time the effect gradually declined (14, 29). This finding suggests that BTX-A may need to be supplemented with a multidisciplinary team (MDT) approach together as part of a rehabilitation program to promote sustained clinical outcomes (30).

Extracorporeal shock wave therapy (ESWT) is a non-invasive, relatively inexpensive treatment that is widely used with post-stroke spasticity. ESWT are widely used in the treatment of kidney stones, urethral stones, urinary stones, and biliary stones by releasing energy into the tissues (31). The mechanism of its application to reduce spasticity is still uncertain. Some researchers have proposed the hypothesis that the high energy delivered by ESWT affects the mechanical properties of the muscle and disrupts the functional link between actin and myosin, therefore relieving muscle spasm (32). Another hypothesis on the mechanism of action of ESWT suggests that ESWT promotes the production of nitric oxide (NO), which is involved in the formation of neuromuscular junctions, neurotransmission, memory formation and synaptic plasticity. In addition, it increases blood supply to tissues and activates growth factors in spastic muscles through angiogenesis (33). Kenmoku T et al. found through animal experiments that low-frequency ESWT inhibits acetylcholine binding to receptors and reduces excitability at the neuromuscular junction, thereby relieving muscle spasm (34). Several studies have reported that ESWT improves post-stroke spasticity and enhances motor performance, and has a promising application (35, 36). Therefore, the addition of ESWT in combination with BTX-A can be considered in order to obtain a sustained therapeutic effect in a timely manner. Although ESWT has been demonstrated as a new technology for PSS treatment in several studies, there are fewer studies on the use of BTX-A in combination with ESWT for the relief of PSS.

Several clinical trials have reported in recent years on the therapeutic efficacy of the combination of BTX-A and ESWT in the treatment of poststroke spasticity. A systematic review of existing clinical randomized controlled trial studies may allow a more precise assessment of their effectiveness. If effectiveness is confirmed, it would help to promote the combined use of BTX-A and ESWT. Therefore, the aim of our study was to systematically evaluate the effect of combined BTX-A and ESWT therapy on poststroke spasticity compared with conventional rehabilitation with or without BTX-A, or other interventions.

## 2 Methods

### 2.1 Retrieval strategy

The protocol was registered in the PROSPERO database (CRD42023476654) and all search results were evaluated according to the PRISMA statement. Extensive searches were performed on databases such as PubMed, Cochrane, Embase, Medline, Web of Science, China National Knowledge Infrastructure, Wan Fang Database, China Biology Medicine disc (CBM), and China Science and Technology Journal Database. The search was conducted from the time of the library’s creation until 20 October 2023 to identify potential studies exploring the effects of ESWT combined with botulinum toxin injections for the treatment of post-stroke spasticity. Chinese search terms were “stroke,” “spasticity,” “hypertonia,” “shock wave therapy,” “extracorporeal shock wave,” “botulinum toxin,” “botulinum toxin,” and “botulinum toxin type A,” etc. English search terms were “Stroke,” “Brain Infarction,” “Muscle Spasticity,” “Spasm,” “Botulinum Toxins, Type A,” “Botulinum Toxins,” “Extracorporeal Shockwave Therapy,” etc. The specific search formula is in Supplementary Table 1.

### 2.2 Study eligibility

We included clinical randomized controlled trials involving patients aged 18 years or older with poststroke spasticity to analyze the efficacy of BTX-A injections combined with shockwave therapy in the treatment of spasticity. Specifically speaking, our intervention criteria for inclusion in the literature needed to include both BTX-A and ESWT. On this basis, there are studies that have also combined conventional rehabilitation at the same time, which are also included in our inclusion. Screening by title, abstract browsing, and full-text reading were used to determine whether articles were included. Criteria for inclusion were developed according to the PICOS principles of Population, Intervention, Comparator, Outcomes and Study designs and are summarized in Table 1. Literature, Meta-analyses, reviews, conferences, case reports, animal experiments and non-randomized controlled trials that did not meet the inclusion criteria were excluded.

**Table 1:** Study inclusion criteria.

Target	Inclusion criteria
Research object	Patients over 18 years of age met the diagnosis of stroke and saw limb spasm
Intervening measure	BTX-A combined with ESWT and conventional rehabilitation
Comparison intervention	Routine rehabilitation with or without botulinum toxin A; routine rehabilitation combined with BTX-A and pseudo-ESWT; Routine rehabilitation combined with BTX-A and ES
Outcome indicator	MAS was the main outcome index, and FAM, PROM, BBS, MBI and VAS scores were the secondary outcome indexes
Research design	RCT

### 2.3 Study selection and data abstraction

Two researchers independently assessed eligibility for inclusion, and disagreements were resolved through a third researcher. Titles and abstracts were screened to identify relevant studies, and then the full text was carefully evaluated. References cited in selected articles were also examined to identify potentially relevant studies. The Modified Ashworth Rating Criteria Score (MAS) was extracted as the primary outcome. Secondary outcomes included simplified Fugl-Meyer scale (FAM) scores, modified Barthel Index (MBI) scores, visual analog scale (VAS), daily spasticity frequency (SFS), passive joint mobility (PROM), and Berg Balance Scale (BBS) scores. Changes in these scores represent certain functional changes.

### 2.4 Quality assessment

The quality of included studies was assessed by the Cochrane Collaboration’s tool for assessing risk of bias, which is recommended for use in systematic reviews of interventions in the Cochrane Handbook version 5.1.0. We assessed seven areas of bias, including selection bias, implementation bias, detection bias, attribution bias, reporting bias, and other sources of bias. Judgments were expressed as “high risk,” “low risk,” or “unclear risk,” and quality assessment data were generated by RevMan version 5.4. The AMSTAR quality assessment form was also used for self-assessment.

## 3 Results

### 3.1 Search results

**Figure 1:**
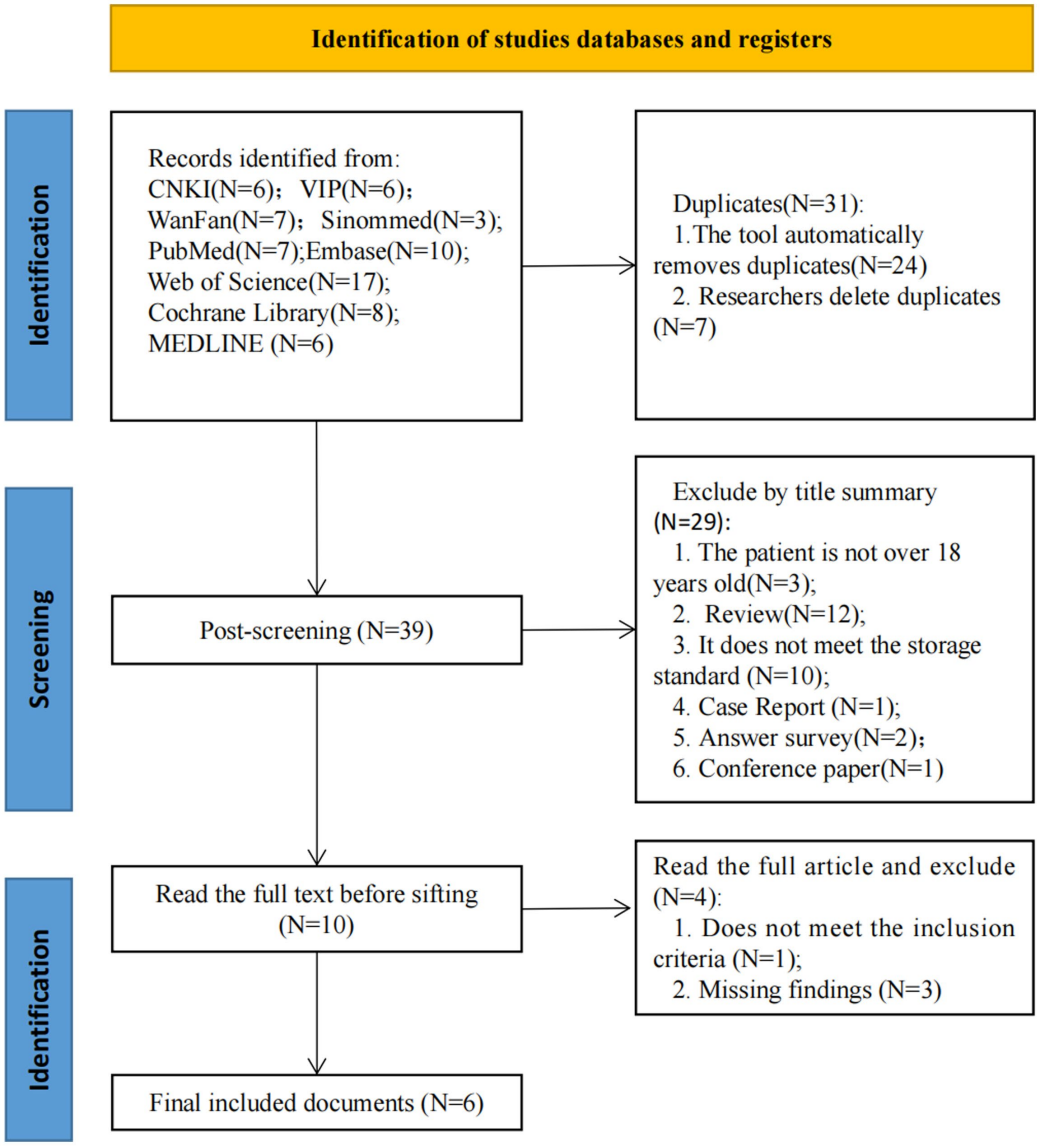
Flowchart.

The initial search produced 70 articles, and 39 records were screened after removing duplicates. 29 records were excluded after assessing titles and abstracts of potentially relevant studies. The full text of 10 articles was then scrutinized, and 4 articles were excluded because they did not meet the inclusion criteria or had missing study results (Figure 1). Finally, 6 RCTs were included in this systematic review (37–42).

### 3.2 Characteristics of included systematic review

The included studies were published from 2013 to the present and contained both English (*N* = 2) (37, 38) and Chinese (*N* = 4) (39–42) literature. Five of them were prospective randomized controlled studies and one was a retrospective randomized controlled study. The characteristics of these studies are displayed in Table 2. Participants’ baseline functional levels are shown in Supplementary Table 2. The sample sizes ranged from 30 to 78. All experimental groups received local injection of botulinum toxin combined with shockwave therapy, while the control group received different treatments, including conventional rehabilitation (*N* = 3) (40–42), conventional rehabilitation combined with botulinum toxin type A (*N* = 2) (37, 42), conventional rehabilitation combined with botulinum toxin injections and pseudo-shockwave therapy (*N* = 1) (39), and botulinum toxin injections combined with electrical stimulation (*N* = 1) (38). The study by Hong Wang et al. (42) included two control groups, conventional rehabilitation combined with or without botulinum toxin. Inclusion and exclusion criteria for each article are recorded in Supplementary Table 3.

**Table 2:** Basic features of included studies.

Included studies	Number	M/F		Average age		Course of disease		Outcomes
	(T/C)	T	C	T	C	T	C	
Megna et al. (37)	15/15	8:7	7:8	58.9 ± 5.4	59.3 ± 6.2	-	-	(1) (4) (8) (9) (10)
Santamato et al. (38)	16/16	7:9	6:10	64.4 ± 6.09	63.1 ± 7.03	10.5 ± 2.12(m)	9.3 ± 3.97(m)	(1) (4) (7)
Lu (41)	39/39	26:13	26:13	59.04 ± 4.43	58.95 ± 4.38	14.82±2.72(d)	14.74±2.67(d)	(1) (2) (4) (6)
Liang et al. (40)	30/30	17:13	16:14	49.93 ± 11.23	50.09 ± 11.07	60.07±11.13 (d)	50.09±11.07(d)	(1) (2) (3)
Duan et al. (39)	18/18	8:10	9:9	51.8 ± 6.2	52.6 ± 7.9	90.5±19.4(d)	87.5±18.7(d)	(1) (2) (3) (5)
Wang et al. (42)	20/20	9:11	11:9^a^	52.18 ± 13.66	51.23±14.24^a^	7.61±3.50(m)	7.98±3.05(m)^a^	(1) (2) (3)
			12:8^b^		50.89±15.16^b^		8.02±3.17(m)^b^	

T is the treatment group; C is the control group; “-” indicates no report; (1) MAS score; (2) FMA score; (3) MBI score; (4) VAS score; (5) PROM; (6) BBS rating; (7) SFS score; (8) muscle tone; (9) Muscle stiffness; (10) Adverse reactions. Wang et al. included two control groups, ^a^indicates the conventional rehabilitation group, ^b^indicates conventional rehabilitation combined with BTX-A; d = day; m = mouth.

The specific treatment parameters for botulinum toxin injections and shockwave therapy for each of the included studies are shown in Table 3. The dose of botulinum toxin used varied from study to study, with the injected dose ranging from 20 to 120 units per muscle group in most studies, and the total dose not exceeding 500 U. In fact, one study had an injection dose of 779.21 ± 5.56/781.35 ± 9.18 U in the control and experimental groups (37). The therapeutic parameters of shock waves used varied from study to study (number of pulses 1,000–3,000, therapeutic pressure 1.5–3 bar, frequency 4–10 Hz). In addition, one study did not mention the frequency of operation of shock wave therapy (37).

**Table 3:** Treatment parameters included in the study.

Included studies	Included studies		Position (U/L)	BTX-A dosage	Shock wave			
	T	C		(T/C)	Pulse count	Treatment pressure	Frequency of treatment	Operation frequency
Megna et al. (37)	BTX-A + ESWT + Routine rehabilitation	BTX-A + Routine rehabilitation	U, L	779.21 ± 5.56/781.35 ± 9.18	1,500 times	1.5 bar	1, 4, 7 days after injection	-
Santamato et al. (38)	BTX-A + ESWT	BTX-A + ES	U	112.4 ± 22.7/118.6 ± 26.4	1,000 times	-	Once /d for 5 consecutive days	4 Hz
Lu (41)	BTX-A + ESWT + Routine rehabilitation	Routine rehabilitation	-	Each group 20-40 U, total dose ≤500 U	3,000 times	1.5 bar	Twice a week for 2 weeks	10 Hz
Liang et al. (40)	BTX-A + ESWT + Routine rehabilitation	Routine rehabilitation	U	The maximum injection volume of each group is 100 U	1,500–2,000 times	2Bar	Twice a week for 6 weeks	10 Hz
Duan et al. (39)	BTX-A + ESWT + Routine rehabilitation	BTX-A + Routine rehabilitation+ pseudo-ESWT	L	The maximum injection volume of each group is 100 U	2,000 times	2-3Bar	1–3 days, once/day; From 2 weeks, once/3days for 4 weeks	8 Hz
Wang et al. (42)	BTX-A + ESWT + Routine rehabilitation	BTX-A + Routine rehabilitation	U, L	Total dose ≤400 U	1,500–2,000 times	2Bar	Once a week for 4 weeks	8 Hz

T is the treatment group; C is the control group; U is the upper lambs; L is the lower limbs; BTX-A, botulinum toxin type A; ESWT, extracorporeal shock wave therapy; ES, electrical stimulation; “-” means not reported.

In the six included articles, the treatment sites for BTX-A injections and shockwaves were as follows: gastrocnemius (37, 39, 42), soleus (39, 42), tibialis posterior (42), bunion (42), phalanges (42), forearm superficial finger flexors (38), biceps brachii (BB) (37), and superficial finger flexors (SFD) (37). In addition, two studies did not report specific treatment sites (40, 41).

### 3.3 Quality assessment and bias analysis

The quality of the included studies was evaluated using the Cochrance Risk of Bias Assessment Tool and the AMSTAR Quality Evaluation Form recommended by the Cochrance Collaborative Group.

**Figure 2:**
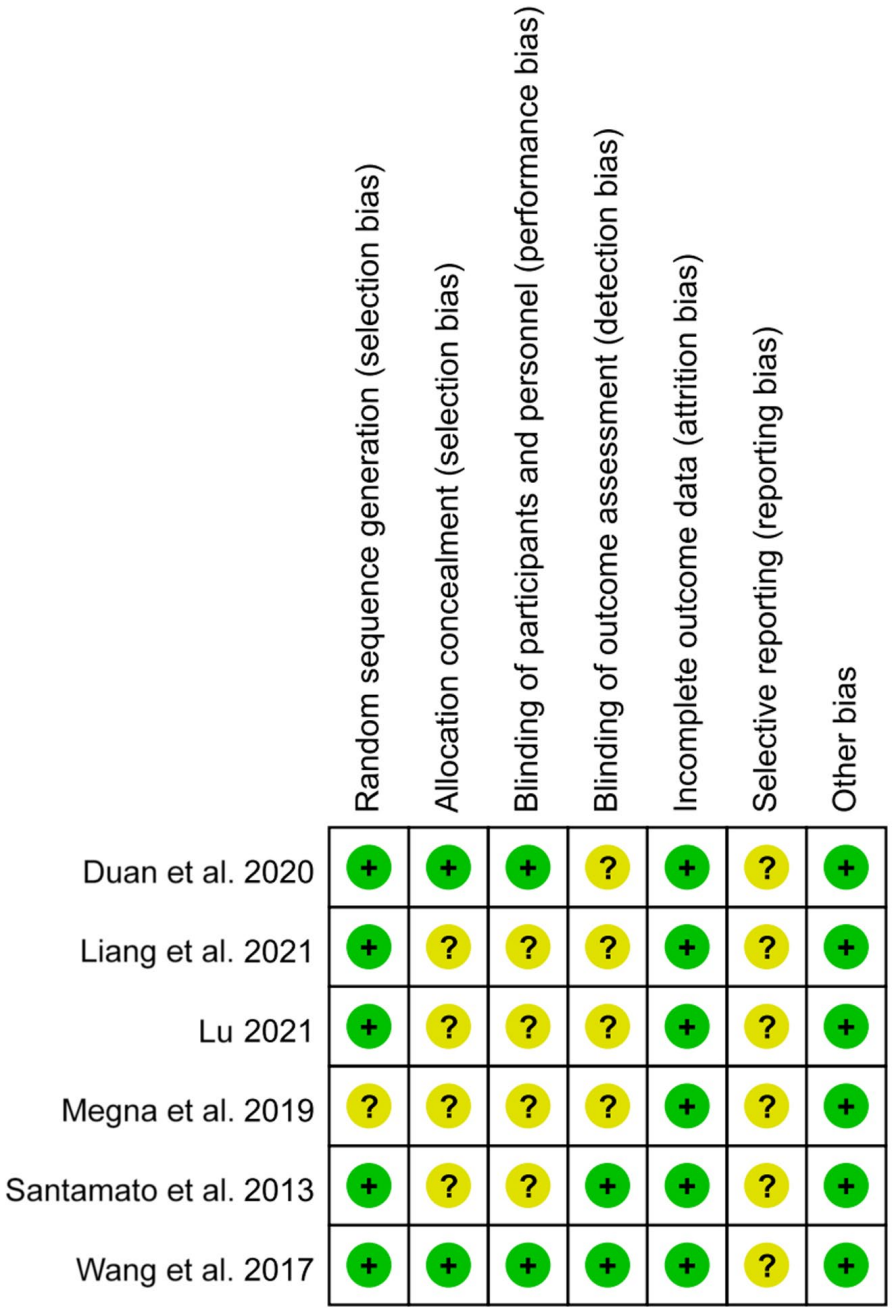
Summary of bias risk: Judgment of each bias risk item for each included study.

**Figure 3:**
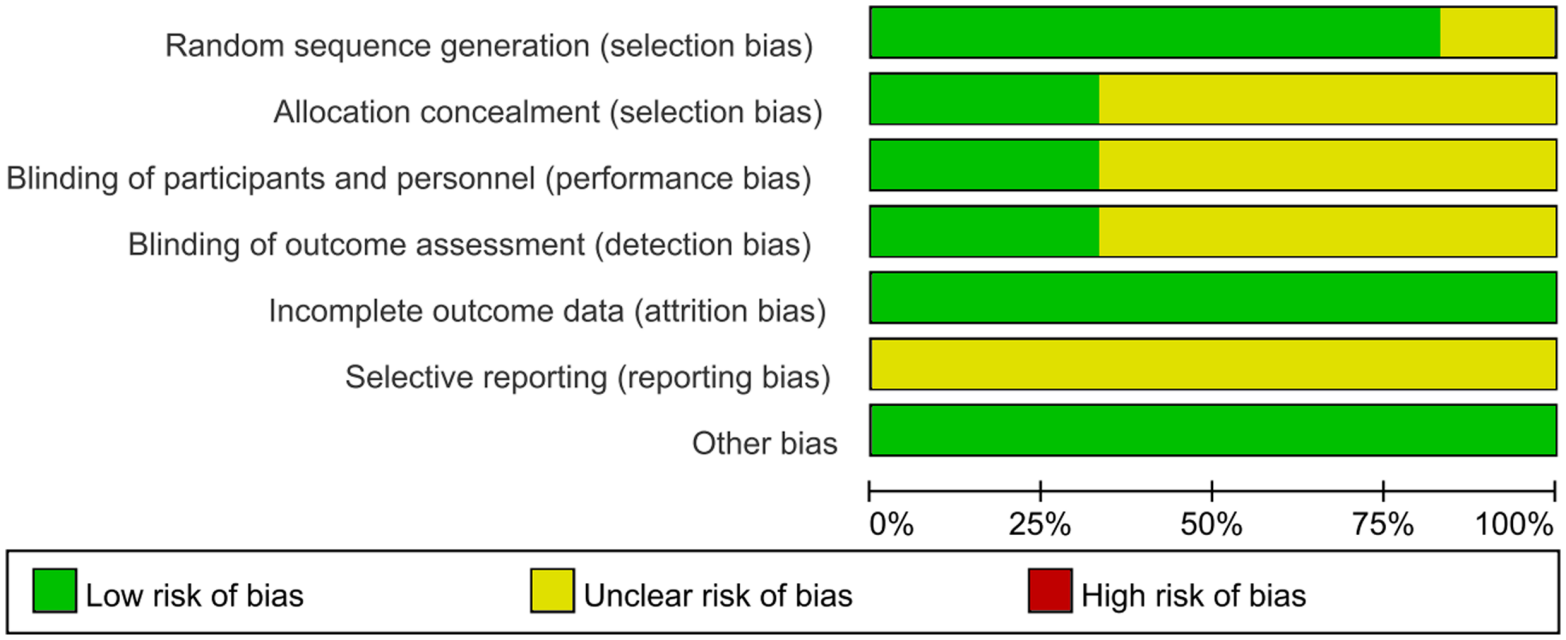
Bias risk graph. The authors’ judgment of each item at risk of bias was expressed as a percentage across all included studies.

The risk of Cochrance bias assessment is shown in Table 4: (1) Random sequence generation: 4 papers used random number table method for random grouping and were judged to be low risk (39–42), 1 paper used software for randomization and was judged to be low risk (38), and the remaining 1 paper only mentioned the word “random” without describing the method of randomization, so it was not possible to judge the risk status (2, 37). (2) Concealment of the allocation plan: 2 articles concealed the allocation plan for patients and were judged to be low risk (39, 42), and 4 included articles did not describe the concealment of the allocation plan (37, 38, 40, 41). (3) Double-blinding of investigators and subjects: 2 were single-blinded but would not affect outcome indicators and were judged to be low risk (39, 42). The remaining 4 papers did not mention blinding and did not have enough information to judge whether they were high or low risk (37, 38, 40, 41). (4) Blinded evaluation of study outcomes: 2 literature assessors were not aware of the subgroups and judged them to be low risk (38, 42), and the remaining 4 were not described and did not have enough information to judge whether they were high or low risk (37, 39–41). (5) Completeness of outcome data: 5 included studies had complete outcome data and were judged to be low risk (37–41), and 1 had an explanation of the lost visit data and could be judged to be low risk (42). (6) Selective publication: There was not enough information to make a judgment of high or low risk. (7) Bias from other sources: None of the studies introduced bias from other sources and were judged to be at low risk (Figures 2, 3).

**Table 4:** Risk assessment of Cochrance bias.

Included studies	Random method	Allocation concealment	Therapeutic blindness	Outcome blind method	Data integrity	Selective publication	Others
Megna et al. (37)	Random	Unclear	Unclear	Unclear	Complete	Unclear	No
Santamato et al. (38)	Digital Photo Professional	Unclear	Unclear	Blind method	Complete	Unclear	No
Lu (41)	Numerical random table method	Unclear	Unclear	Unclear	Complete	Unclear	No
Liang et al. (40)	Numerical random table method	Unclear	Unclear	Unclear	Complete	Unclear	No
Duan et al. (39)	Numerical random table method	Centralized control	Single-blind	Unclear	Complete	Unclear	No
Wang et al. (42)	Numerical random table method	Centralized control	Single-blind	Blind method	One case was lost to follow-up	Unclear	No

The quality evaluation is strictly in accordance with the requirements of the AMSTAR quality evaluation form for systematic evaluation, strict quality control, eligible, the results are shown in Table 5.

**Table 5:** AMSTAR quality evaluation form.

Item	Option			
1. Whether the system review has been carefully designed in advance	Yes	No	Cannot answer	Not applicable
2. Whether more than two people have completed literature screening and data extraction	Yes	No	Cannot answer	Not applicable
3. Whether the literature search has been conducted comprehensively and systematically	Yes	No	Cannot answer	Not applicable
4. Whether publication types (e.g., gray literature) were used as inclusion criteria	Yes	No	Cannot answer	Not applicable
5. Is a list of references (inclusion and exclusion) available	Yes	No	Cannot answer	Not applicable
6. Whether the basic features of the included literature are provided and described	Yes	No	Cannot answer	Not applicable
7. Whether the quality of the included literature was rigorously evaluated	Yes	No	Cannot answer	Not applicable
8. Whether the results of literature quality evaluation were used to form final conclusions	Yes	No	Cannot answer	Not applicable
9. Whether the method of summary analysis is appropriate	Yes	No	Cannot answer	Not applicable
10. Whether the possibility of publication bias was assessed	Yes	No	Cannot answer	Not applicable
11. Whether potential conflicts of interest have been declared	Yes	No	Cannot answer	Not applicable

### 3.4 Outcome

#### 3.4.1 Spasticity

Modified Ashworth Scale (MAS) is a commonly used scale for clinical evaluation of patients’ muscle tone and assessing the degree of muscle spasticity (43). The MAS classifies the degree of spasticity into grades 0-IV: (1) Grade 0: normal muscle tone; (2) Grade I: a slight increase in muscle tone, with minimal resistance at the end of the joint movement; (3) Grade I+: a slight increase in muscle tone, with minimal resistance in the posterior 50% of the joint range of motion; (4) Grade II: an increase in muscle tone in the majority of the joint range of motion, but still able to carry out passive activities; (5) Grade III: a significant increase in muscle tone, with difficulty in passive activities; (6) Grade IV: stiffness occurs and inactivity is not possible (44). This measure is reliable by assessing the degree of resistance and the point of resistance during manual stretching of the muscle (45). The outcome indicators varied among the studies. However, the included studies all used the MAS to subjectively report treatment outcomes. The results showed that on the basis of conventional rehabilitation and BTX-A, the efficacy of supplemental ESWT therapy for spasticity relief was better than the control group, and the differences were statistically significant (*p* < 0.05) (37–42).

In addition, Megna et al. performed an objective assessment of muscle tone by MyotonPro®. One month after the treatment, muscle tone was significantly reduced (pre = 29.11 ± 1.43, 1 M = 20.01 ± 1.54) and was significantly different from the control group (pre = 28.34 ± 1.23; 1 M = 22.20 ± 1.56). The study also evaluated muscle hardness. Compared with the control group (pre = 312.89 ± 4.25, 1 M = 212.34 ± 1.34), ESWT supplementation treatment (pre = 311.65 ± 2.43, 1 M = 204.81 ± 2.21) reduced muscle hardness more effectively (*p* < 0.05) (37).

One study evaluated the daily spasm frequency scale (SFS). SFS is generally recorded by the patients themselves as the number of spasms occurring per hour or per day (46). Santamato et al. categorized 32 patients into either a shockwave group or an electrical stimulation (ES) group. Both groups received conventional rehabilitation and injections of BTX-A. The results showed that the combination of BTX-A and f-ESWT had a better effect on the reduction of spasticity grade (*p* < 0.05) (38).

#### 3.4.2 Athletic ability

The simplified Fugl-Meyer assessment (FMA) has an excellent retest reliability and is internationally recognized (47). The FMA is based on Twitchell and Brunnstrom’s observations of the sequential recovery of motor function, assessing reflex activity of the upper and lower extremities as well as synergistic and dissociative movements (48). It is the most widely used quantitative measure of motor recovery after stroke (49). Four studies have reported the results of FMA scores, which showed that BTX-A injection followed by combined ESWT therapy improved patients’ upper or lower extremity motor function (*p* < 0.05) (39–42).

The results of the study by Duan et al. showed that the MAS score and PROM of the observation group were significantly better than those of the control group after 2 and 4 weeks of treatment (*p* < 0.05). This suggests that BTX-A combined with low-frequency ESWT can more effectively alleviate the spasticity state of the calf triceps muscle after stroke and improve the joint mobility. The FMA and MBI scores of the observation group were also significantly better than those of the control group after 4 weeks of treatment (*p* < 0.05). This suggests that compared with local injection of BTX-A alone, BTX-A combined with ESWT treatment did not significantly improve the motor function and ADL ability of the affected lower limb in the short term (2 weeks). Rather, it was only after a longer period of regular rehabilitation training (4 weeks) that the observation group significantly outperformed the control group in terms of motor function and ADL ability. This also suggests that the purpose of BTX-A injection and low-frequency ESWT is not only to alleviate muscle spasm, but also to promote the recovery of limb motor function through active exercise based on the reduction of muscle tone (39).

One study evaluated balance function. The Berg balance scale (BBS) has a total of 14 items, each with five levels ranging from 0 to 4 out of a possible 56 points. The total score ultimately reflects the overall balance ability of the subject. The higher the score, the better the balance (50). Lu recruited 78 patients for a 2-week treatment and assessed the patients’ balance ability before (32.23 ± 3.62) and 2 weeks after (42.65 ± 4.50) the treatment. The results suggested that the combination therapy was able to improve the balance of the patients (*p* < 0.05) (41).

A study evaluated passive range of motion (PROM). Duan et al. performed botulinum toxin injections in patients with post-stroke triceps spasticity of the calf and gave shockwave therapy 1–3 days post-injection, once/day, and from week 2 onwards, once/3 days for 4 weeks. The passive knee mobility of the patients was assessed before (25.58 ± 4.52), 2 weeks (44.37 ± 8.02) and 4 weeks (56.92 ± 8.79) after the treatment. The results indicated that the combination therapy was able to improve the contracture status of the joint (*p* < 0.05). Moreover, shockwave therapy was able to improve the passive joint mobility of patients in this study compared to the pseudo-ESWT group (pre = 27.02 ± 4.39, 2 W = 36.74 ± 6.75, 4 W = 45.48 ± 7.86; *p* < 0.05) (39).

#### 3.4.3 Activity of daily living

The modified Barthel index (MBI) is a commonly used scale to assess the ability to perform activities of daily living (ADL; including eating, grooming, bathing, toileting, dressing, diaphragm control, postural transfer, and walking up and down stairs). 0–100 points are scored, with higher scores indicating better ADL ability of patients (51). Three studies reported the results of Modified Barthel Index (MBI) scores, which suggested that the combined management of conventional rehabilitation, BTX-A and ESWT therapies could improve patients’ self-care ability (*p* < 0.05) (39, 40, 42).

#### 3.4.4 Pain

The Visual Analog Scale (VAS) is one of the most commonly used unidimensional measures of pain intensity and is characterized by accuracy, simplicity, and sensitivity (52). Three studies have assessed patients’ self-perceived pain before and after treatment by VAS scores, demonstrating that combination therapy can reduce patients’ pain (37, 38, 41).

#### 3.4.5 Adverse event

The combined management of conventional rehabilitation, BTX-A and ESWT therapy may have a favorable safety profile, with only one study reporting adverse events. One case (3.3%) in the experimental group reported localized muscle weakness with a duration of 7 days. The control group reported one case (3.3%) of localized muscle weakness and one case (3.3%) of transient generalized weakness (37).

#### 3.4.6 Follow-up outcome

Three studies reported follow-up results. Megna et al. performed shockwave therapy on days 1, 4, and 7 after BTX-A injection and followed up. In this study, during follow-up, an increase in MAS scores was found in patients at 2 months (2.4 ± 0.6) and 3 months (3.2 ± 0.2) compared to 1 month post-treatment (1.5 ± 0.25), and the same trend was observed for muscle tone (1 M =20.01 ± 1.54,2 M =24.87 ± 1.78, 3 M =28.99 ± 1.11), muscle hardness (1 M = 204.81 ± 2.21, 2 M = 240.31 ± 2.31, 3 M = 309.12 ± 3.89), and VAS scores (1 M = 5.0 ± 0.6, 2 M = 6.10 ± 0.9, 3 M = 8.8 ± 0.5) (37). The frequency of shockwave therapy used by Santamato et al. was 1x/d for 5d after BTX-A injection. Spasticity grade increased at 30d (1.75 ± 0.45) and 90d (1.58 ± 0.52) follow-up compared to 15d post-treatment (1.37 ± 0.5) (38). The study by Wang et al. provided similar conclusions, with an increase in spasticity (1 M = 1.45 ± 0.16, 4 M = 2.14 ± 0.19) and a decrease in motor function (1 M = 28.56 ± 1.56, 4 M = 19.21 ± 3.09) and ADL (1 M = 58.29 ± 5.32, 4 M = 49.83 ± 5.41) found in the patients during follow-up (42). This may suggest that we need to consider the appropriate frequency of shockwave therapy as well as the treatment period for long-term spasticity management.

## 4 Discussion

Post-stroke spasticity (PSS) is defined as involuntary muscle activity, which can be caused by intermittent or persistent motor-sensory control deficits. PSS is secondary to damage to the upper motor elements and is a manifestation of the gradual recovery of the function of the body’s pyramidal tracts (53). However, PSS can lead to contractual deformities accompanied by pain and limited joint movement, limiting patients’ daily activities and reducing their quality of life (54). For focal spasticity, BTX injections are considered the most effective and safest treatment strategy (55). Although highly utilized, the efficacy of BTX-A may decrease after the end of the application cycle, and compensation by increasing the dose has limited effects in terms of time and cost (56). And some studies have found limited efficacy against focal lower limb spastic dyskinesia in adults (57). ESWT has been shown to be effective in reducing pain, improving muscle spasm and enhancing limb function in stroke patients. Consider applying ESWT as a complementary therapy after BTX-A injections for sustained anti-spasticity effects.

Previous systematic reviews and meta-analyses only evaluated the efficacy of BTX-A or ESWT alone in PSS (58) or compared the efficacy of the two (33, 59), and no systematic reviews elaborating on the efficacy of the combination were found. In recent years, there has been an increase in the number of randomized clinical trials of BTX-A in combination with ESWT for the treatment of PSS, with two studies in progress (60, 61). This has made it possible to observe the benefits of BTX-A in combination with ESWT in clinical practice. Six randomized controlled trials with a total of 296 patients were included in this study. The differences in MAS scores (*p* < 0.05), FMA (*p* < 0.05), MBI scores (*p* < 0.05), and VAS scores (*p* < 0.05) were statistically significant after treatment with the combination therapy of BTX-A with ESWT as compared to the control group. Therefore, BTX-A combined with ESWT not only relieved the state of muscle spasm, but also promoted the recovery of limb motor function based on the reduction of muscle tone and alleviated patients’ pain. In addition, some studies have found inconsistencies between BTX-A and ESWT in terms of time to improvement in spasticity and improvement in motor function.

Regarding the degree of spasticity, all articles used the MAS score as an outcome indicator. BTX-A combined with ESWT therapy effectively reduced patients’ MAS scores and relieved the degree of spasticity in the short term (4 W) compared with pre-treatment. Whether compared with conventional rehabilitation or conventional rehabilitation combined with BTX-A or BTX-A combined with ES, BTX-A combined with ESWT relieved spasticity more effectively. In addition to this, the short-term efficacy of the combination therapy was supported by an objective assessment of muscle tone by MyotonPro® by Megna et al. (37). However, there were 2 studies reporting follow-up results, and we found limited long-term (3 M) efficacy of BTX-A combined with ESWT for spasticity relief. This may also be related to the frequency and length of treatment with ESWT. Specifically: Megna et al. (37) performed ESWT on days 1, 4, and 7 after BTX-A injection at a treatment pressure of 1.5 bar. Santamato et al. (38) used an ESWT frequency of 1x/d after BTX-A injection for 5 days at 4 Hz. Another study reporting follow-up results reported different results. Wang et al. (42) showed an increase in spasticity at 4 M follow-up, but still had significant spasticity relief compared to pre-treatment. In this study, ESWT was administered once a week for 4 weeks at a pressure of 2 bar and a frequency of 8 Hz. In addition, Lu et al. used ESWT twice a week for a total of 2 weeks. Duan et al. (39) treated once a day for 1–3 days after BTX-A injection, and then changed to once every 3 days from the second week onwards for a total of 4 weeks. However, both studies only reported short-term efficacy without long-term follow-up. Liang et al. (40) used twice-weekly treatment for a total of 6 weeks. A decrease in spasticity was observed at the endpoint (6 W).

Similarly, BTX-A in combination with ESWT therapy has been shown to be effective in improving patients’ upper or lower extremity mobility. It has also been shown that the combination therapy improves balance function of the lower extremities and increases passive joint mobility. However, Duan et al. (39) found that the combination of BTX-A and ESWT therapy was inconsistent in reducing spasticity and improving motor function over time. BTX-A combined with ESWT therapy did not significantly improve lower extremity motor function and ADL ability on the affected side at 2 weeks. Rather, it was only after 4 weeks of ESWT treatment and regular rehabilitation that the observation group significantly outperformed the control group in terms of motor function and ADL ability. Only one study followed up motor function, which had the same trend as the degree of limb spasticity in Wang et al. et al. (42). And at 4 M post-treatment, motor and ADL abilities were significantly better compared to pre-treatment.

In addition to this, BTX-A combined with ESWT therapy is also effective in mentioning pain relief. However, two studies showed discrepancies in the follow-up results for pain. Megna et al. (37) reported VAS scores (Pre = 8.9 ± 0.3; 3 M = 8.8 ± 0.5) and Santamato et al. (38) reported VAS scores (Pre = 5 ± 1.21; 3 M = 1.87 ± 0.62). Regarding the safety of combination therapy, only the study by Megna et al. (37) reported adverse events. In this study, conventional rehabilitation combined with BTX-A was used in the control group and conventional rehabilitation combined with BTX-A and ESWT was used in the experimental group. Symptoms of muscle weakness were observed in both groups. No related adverse events were reported in other studies. In conclusion BTX-A combined with ESWT therapy has a good safety profile.

Moreover, differences in BTX-A dosage may affect the efficacy analysis of the combination therapy. The specific doses of BTX-A are shown in Table 3. Unfortunately, we were unable to characterize the role of BTX-A dosage in relieving spasticity and improving movement. This is because this review focuses on the efficacy of the combination therapy of BTX-A and ESWT. In some studies, BTX-A was administered in both test and control groups. And the injection site varied from study to study, and the description of the dose was not uniform. In conclusion, the combination therapy of BTX-A and ESWT can effectively relieve spasticity in patients with PSS, improve locomotion and activities of daily living, and relieve patients’ pain.

ESWT has shown promising utility in the clinic, meaningfully reducing spasticity and improving motor function. However, there are no standardized treatment parameters for ESWT for spasticity, including ESWT intensity, frequency, number of pulses, and session duration. To ensure the effectiveness of the treatment, more in-depth clinical studies are needed in the future in order to incorporate ESWT into an effective approach for comprehensive stroke rehabilitation programs and to establish evidence-based guidelines for its application. For patients receiving BTX-A injections, the appropriate timing of ESWT interventions could be further explored. Determining the optimal timing and periodicity of ESWT interventions can help maximize the benefits of ESWT and promote overall stroke recovery. Given that the quality of current studies of combination therapy is not optimal, rigorous study designs, validated spasticity assessment tools, and relevant animal studies are expected to provide more substantial scientific evidence for combination therapy.

## 5 Limitation

This review has some limitations in terms of the completeness of the literature search. First, the search only included literature in Chinese and English, which may have omitted potentially relevant articles in other languages. Second, the number of included literature was small, and therefore the results of the studies were unstable. The included studies were small and lacked data from large studies to support the efficacy of combination therapy. Also, treatment doses and parameters were not the same across studies. Finally, the method of bias analysis in this paper is somewhat subjective. More clinical trials are needed to supplement this in the future.

## 6 Conclusion

Management of spasticity states remains challenging. Increasing attention is being paid to the contribution of combining anti-spasticity medications with nonpharmacologic interventions to early and long-term spasticity management. Because high-quality studies of BTX-A in combination with ESWT for spasticity are still insufficient, further studies with well-designed experiments are needed to finalize the most effective management of spasticity status. Future studies could focus on the dose of the combined intervention, frequency of treatment, and treatment period to develop effective spasticity management and improve long-term functional outcomes after stroke. Given the current published evidence, the combination of BTX-A and ESWT is critical for providing long-term, comprehensive rehabilitation for spasticity states after stroke.

## Data availability statement

The original contributions presented in the study are included in the article/Supplementary material, further inquiries can be directed to the corresponding author.

## Author contributions

Y-nD: Writing – original draft, Writing – review & editing. YL: Writing – original draft, Writing – review & editing. T-yZ: Writing – original draft, Writing – review & editing. NJ: Writing – original draft, Writing – review & editing. H-yD: Writing – original draft, Writing – review & editing.
