# Supplementary material for: Efficacy of botulinum toxin A combined with extracorporeal shockwave therapy in post-stroke spasticity: a systematic review
Source: Front Neurol. 2024 Mar 15;15:1342545. doi: 10.3389/fneur.2024.1342545 (PMC10979702; doi:10.3389/fneur.2024.1342545)
Supplement: Supplementary file 1 [file Data_Sheet_1.docx]

Supplementary table 1 Search formula

| Serial No. | Retrieval formula |
| --- | --- |
| #1 | “Stroke” OR “Strokes” OR “Cerebrovascular Accident” OR “Cerebrovascular Accidents” OR “CVA (Cerebrovascular Accident)” OR “CVAs (Cerebrovascular Accident)” OR “Cerebrovascular Apoplexy” OR “Apoplexy, Cerebrovascular” OR “Vascular Accident, Brain” OR “Brain Vascular Accident” OR “Brain Vascular Accidents” OR “Vascular Accidents, Brain” OR “Cerebrovascular Stroke” OR “Cerebrovascular Strokes” OR “Stroke, Cerebrovascular” OR “Strokes, Cerebrovascular” OR “Apoplexy” OR “Cerebral Stroke” OR “Cerebral Strokes” OR “Stroke, Cerebral” OR “Strokes, Cerebral” OR “Stroke, Acute” OR “Acute Stroke” OR “Acute Strokes” OR “Strokes, Acute” OR “Cerebrovascular Accident, Acute” OR “Acute Cerebrovascular Accident” OR “Acute Cerebrovascular Accidents” OR “Cerebrovascular Accidents, Acute” |
| #2 | “Brain Infarction” OR “Brain Infarctions” OR “Infarction, Brain” OR “Infarctions, Brain” OR “Brain Infarct” OR “Brain Infarcts” OR “Infarct, Brain” OR “Infarcts, Brain” OR “Anterior Circulation Brain Infarction” OR “Infarction, Brain, Anterior Circulation” OR “Infarction, Anterior Circulation, Brain” OR “Anterior Circulation Infarction, Brain” OR “Brain Infarction, Anterior Circulation” OR “Venous Infarction, Brain” OR “Brain Venous Infarction” OR “Brain Venous Infarctions” OR “Infarction, Brain Venous” OR “Infarctions, Brain Venous” OR “Venous Infarctions, Brain” OR “Brain Infarction, Venous” OR “Brain Infarctions, Venous” OR “Infarction, Venous Brain” OR “Infarctions, Venous Brain” OR “Venous Brain Infarction” OR “Venous Brain Infarctions” OR “Anterior Cerebral Circulation Infarction” OR “Infarction, Anterior Cerebral Circulation” OR “Brain Infarction, Posterior Circulation” OR “Posterior Circulation Infarction, Brain” OR “Posterior Circulation Brain Infarction” OR “Infarction, Brain, Posterior Circulation” OR “Infarction, Posterior Circulation, Brain” |
| #3 | “Infarction, Posterior Circulation, Brain” OR “Brain Stem Infarction” OR “Infarction, Brain Stem” OR “Infarctions, Brainstem” OR “Brainstem Stroke” OR “Stroke, Brainstem” OR “Infarctions, Brain Stem” OR “Brain Stem Infarct” OR “Brain Stem Infarcts” OR “Infarct, Brain Stem” OR “Infarcts, Brain Stem” OR “Stem Infarct, Brain” OR “Stem Infarcts, Brain” OR “Brainstem Infarctions” OR “Brainstem Infarction” OR “Infarction, Brainstem” OR “Claude Syndrome” OR “Weber Syndrome” OR “Millard-Gublar Syndrome” OR “Millard Gublar Syndrome” OR “Syndrome, Millard-Gublar” OR “Top of the Basilar Syndrome” OR “Benedict Syndrome” OR “Foville Syndrome” |
| #4 | “Lateral Medullary Syndrome” OR “Lateral Medullary Syndromes” OR “Syndromes, Lateral Medullary” OR “Posterior Inferior Cerebellar Artery Syndrome” OR “Wallenberg's Syndrome” OR “Wallenbergs Syndrome” OR “Vieseaux-Wallenberg Syndrome” OR “Syndrome, Vieseaux-Wallenberg” OR “Vieseaux Wallenberg Syndrome” OR “Wallenberg Syndrome” OR “Syndrome, Wallenberg” OR “Dorsolateral Medullary Syndrome” OR “Medullary Syndrome, Dorsolateral” OR “Lateral Bulbar Syndrome” |
| #5 | “Cerebral Infarction” OR “Cerebral Infarctions” OR “Infarctions, Cerebral” OR “Infarction, Cerebral” OR “Cerebral Infarct” OR “Cerebral Infarcts” OR “Infarct, Cerebral” OR “Infarcts, Cerebral” OR “Cerebral Infarction, Left Hemisphere” OR “Left Hemisphere, Infarction, Cerebral” OR “Infarction, Left Hemisphere, Cerebral” OR “Left Hemisphere, Cerebral Infarction” OR “Cerebral, Left Hemisphere, Infarction” OR “Infarction, Cerebral, Left Hemisphere” OR “Subcortical Infarction” OR “Infarction, Subcortical” OR “Infarctions, Subcortical” OR “Subcortical Infarctions” OR “Posterior Choroidal Artery Infarction” OR “Anterior Choroidal Artery Infarction” OR “Cerebral Infarction, Right Hemisphere” OR “Right Hemisphere, Cerebral Infarction” OR “Infarction, Right Hemisphere, Cerebral” OR “Right Hemisphere, Infarction, Cerebral” OR “Cerebral, Right Hemisphere, Infarction” OR “Infarction, Cerebral, Right Hemisphere” |
| #6 | “CADASIL” OR “Dementia, Hereditary Multi-Infarct Type” OR “Dementia, Hereditary Multi Infarct Type” OR “Cerebral Autosomal Dominant Arteriopathy with Subcortical Infarcts and Leukoencephalopathy” OR “Cerebral Arteriopathy with Subcortical Infarcts and Leukoencephalopathy” OR “CADASILM” |
| #7 | “Dementia, Multi-Infarct” OR “Dementia, Multi Infarct” OR “Dementias, Multi-Infarct” OR “Multi-Infarct Dementias” OR “Dementia Multi-Infarct” OR “Dementia Multi Infarct” OR “Dementia Multi-Infarcts” OR “Multi-Infarct, Dementia” OR “Multi-Infarcts, Dementia” OR “Dementia, Multiinfarct” OR “Dementias, Multiinfarct” OR “Multiinfarct Dementia” OR “Multiinfarct Dementias” OR “Multi-Infarct Dementia” OR “Multi Infarct Dementia” OR “Lacunar Dementia” OR “Dementia, Lacunar” OR “Dementias, Lacunar” OR “Lacunar Dementias” |
| #8 | “Infarction, Anterior Cerebral Artery” OR “Anterior Cerebral Artery Infarction” OR “Anterior Cerebral Artery Stroke” OR “Infarction, Anterior Cerebral Artery Distribution” OR “Stroke, Anterior Cerebral Artery” OR “ACA Infarct” OR “ACA Infarcts” OR “Infarct, ACA” OR “Infarcts, ACA” OR “ACA Infarction” OR “ACA Infarctions” OR “Infarction, ACA” OR “Infarctions, ACA” OR “Infarction, Anterior Cerebral Artery Circulation” OR “Heubner Artery Infarction” OR “Artery Infarction, Heubner” OR “Infarction, Heubner Artery” OR “Heubner's Artery Infarction” OR “Artery Infarction, Heubner's” OR “Heubners Artery Infarction” OR “Infarction, Heubner's Artery” OR “Anterior Cerebral Artery Syndrome” OR “Syndrome, Anterior Cerebral Artery” |
| #9 | “Infarction, Middle Cerebral Artery” OR “MCA Infarction” OR “Cerebral Infarction, Middle Cerebral Artery” OR “Middle Cerebral Artery Infarction” OR “Middle Cerebral Artery Stroke” OR “Stroke, Middle Cerebral Artery” OR “MCA Infarct” OR “Infarct, MCA” OR “Infarcts, MCA” OR “MCA Infarcts” OR “Middle Cerebral Artery Circulation Infarction” OR “Left Middle Cerebral Artery Infarction” OR “Middle Cerebral Artery Embolus” OR “Embolus, Middle Cerebral Artery” OR “Middle Cerebral Artery Occlusion” OR “Occlusion, Middle Cerebral Artery” OR “Thrombotic Infarction, Middle Cerebral Artery” OR “Middle Cerebral Artery Thrombotic Infarction” OR “Middle Cerebral Artery Thrombosis” OR “Thrombosis, Middle Cerebral Artery” OR “Right Middle Cerebral Artery Infarction” OR “Embolic Infarction, Middle Cerebral Artery” OR “Middle Cerebral Artery Embolic Infarction” OR “Middle Cerebral Artery Syndrome” |
| #10 | “Infarction, Posterior Cerebral Artery” OR “Stroke, Posterior Cerebral Artery” OR “Posterior Cerebral Artery Infarction” OR “Posterior Cerebral Artery Stroke” OR “PCA Infarct” OR “Infarct, PCA” OR “Infarcts, PCA” OR “PCA Infarcts” OR “PCA Infarction” OR “Infarction, PCA” OR “Posterior Cerebral Artery Syndrome” OR “Thrombotic Infarction, Posterior Cerebral Artery” OR “Posterior Cerebral Artery Thrombotic Infarction” OR “Embolic Infarction, Posterior Cerebral Artery” OR “Posterior Cerebral Artery Embolic Infarction” |
| #11 | “Hemorrhagic Stroke” OR “Hemorrhagic Strokes” OR “Stroke, Hemorrhagic” OR “Subarachnoid Hemorrhagic Stroke” OR “Hemorrhagic Stroke, Subarachnoid” OR “Stroke, Subarachnoid Hemorrhagic” OR “Subarachnoid Hemorrhagic Strokes” OR “Intracerebral Hemorrhagic Stroke” OR “Hemorrhagic Stroke, Intracerebral” OR “Intracerebral Hemorrhagic Strokes” OR “Stroke, Intracerebral Hemorrhagic” OR “Intracerebral Hemorrhage Stroke” OR “Hemorrhage Stroke, Intracerebral” OR “Intracerebral Hemorrhage Strokes” OR “Stroke, Intracerebral Hemorrhage” |
| #12 | “Ischemic Stroke” OR “Ischemic Strokes” OR “Stroke, Ischemic” OR “Ischaemic Stroke” OR “Ischaemic Strokes” OR “Stroke, Ischaemic” OR “Cryptogenic Ischemic Stroke” OR “Cryptogenic Ischemic Strokes” OR “Ischemic Stroke, Cryptogenic” OR “Stroke, Cryptogenic Ischemic” OR “Cryptogenic Stroke” OR “Cryptogenic Strokes” OR “Stroke, Cryptogenic” OR “Cryptogenic Embolism Stroke” OR “Cryptogenic Embolism Strokes” OR “Embolism Stroke, Cryptogenic” OR “Stroke, Cryptogenic Embolism” OR “Wake-up Stroke” OR “Stroke, Wake-up” OR “Wake up Stroke” OR “Wake-up Strokes” OR “Acute Ischemic Stroke” OR “Acute Ischemic Strokes” OR “Ischemic Stroke, Acute” OR “Stroke, Acute Ischemic” |
| #13 | “Embolic Stroke” OR “Embolic Strokes” OR “Stroke, Embolic” OR “Strokes, Embolic” OR “Cardioembolic Stroke” OR “Cardioembolic Strokes” OR “Stroke, Cardioembolic” OR “Strokes, Cardioembolic” OR “Cardio-embolic Stroke” OR “Cardio embolic Stroke” OR “Cardio-embolic Strokes” OR “Stroke, Cardio-embolic” OR “Strokes, Cardio-embolic” |
| #14 | “Thrombotic Stroke” OR “Stroke, Thrombotic” OR “Thrombotic Strokes” OR “Acute Thrombotic Stroke” OR “Acute Thrombotic Strokes” OR “Stroke, Acute Thrombotic” OR “Thrombotic Stroke, Acute” |
| #15 | “Stroke, Lacunar” OR “Lacunar Stroke” OR “Lacunar Strokes” OR “Strokes, Lacunar” OR “Lacunar Syndrome” OR “Lacunar Syndromes” OR “Syndrome, Lacunar” OR “Syndromes, Lacunar” OR “Infarction, Lacunar” OR “Infarctions, Lacunar” OR “Lacunar Infarction” OR “Lacunar Infarctions” OR “Infarct, Lacunar” OR “Infarcts, Lacunar” OR “Lacunar Infarct” OR “Lacunar Infarcts” |
| #16 | “#1 OR #2 OR #3 OR #4 OR #5 OR #6 OR #7 OR #8 OR #9 OR #10 OR #11 OR #12 OR #13 OR #14 OR #15” |
| #17 | “Muscle Spasticity” OR “Spasticity, Muscle” OR “Spastic” OR “Clasp-Knife Spasticity” OR “Clasp Knife Spasticity” OR “Spasticity, Clasp-Knife” |
| #18 | “Spasm” OR “Spasms” OR “Muscle Spasm” OR “Muscle Spasms” OR “Spasm, Muscle” OR “Spasms, Muscle” OR “Muscular Spasm” OR “Muscular Spasms” OR “Spasm, Muscular” OR “Spasms, Muscular” OR “Spasm, Generalized” OR “Generalized Spasm” OR “Generalized Spasms” OR “Spasms, Generalized” OR “Spasm, Ciliary Body” OR “Ciliary Body Spasm” OR “Ciliary Body Spasms” OR “Spasms, Ciliary Body” |
| #19 | “Muscle Hypertonia” OR “Hypertonia, Muscle” OR “Hypertonias, Muscle” OR “Muscle Hypertonias” OR “Muscle Tone Increased” OR “Increased, Muscle Tone” OR “Tone Increased, Muscle” OR “Muscular Hypertonicity” OR “Hypertonicities, Muscular” OR “Hypertonicity, Muscular” OR “Muscular Hypertonicities” OR “Hypermyotonia” OR “Hypermyotonias” OR “Hypertonia, Transient” OR “Hypertonias, Transient” OR “Transient Hypertonia” OR “Transient Hypertonias” OR “Hypertonia, Neonatal” OR “Hypertonias, Neonatal” OR “Neonatal Hypertonia” OR “Neonatal Hypertonias” OR “Hypertonia, Sphincter” OR “Hypertonias, Sphincter” OR “Sphincter Hypertonia” OR “Sphincter Hypertonias” OR “Hypertonia, Detrusor Muscle” OR “Detrusor Muscle Hypertonia” OR “Detrusor Muscle Hypertonias” OR “Hypertonias, Detrusor Muscle” OR “Muscle Hypertonia, Detrusor” OR “Muscle Hypertonias, Detrusor” OR “Hypertonia, Infantile” OR “Hypertonias, Infantile” OR “Infantile Hypertonia” OR “Infantile Hypertonias” |
| #20 | “Muscle Rigidity” OR “Rigidity, Muscle” OR “Rigidity, Muscular” OR “Muscular Rigidity” OR “Cogwheel Rigidity” OR “Cogwheel Rigidities” OR “Rigidities, Cogwheel” OR “Rigidity, Cogwheel” OR “Nuchal Rigidity” OR “Rigidity, Nuchal” OR “Extrapyramidal Rigidity” OR “Rigidity, Extrapyramidal” OR “Gegenhalten” OR “Gegenhaltens” OR “Catatonic Rigidity” OR “Rigidity, Catatonic” OR “Extensor Rigidity” OR “Rigidity, Extensor” |
| #21 | #17 OR #18 OR #19 OR #20 |
| #22 | “Botulinum Toxins, Type A” OR “Clostridium botulinum A Toxin” OR “Botulinum Toxin A” OR “Toxin A, Botulinum” OR “Botulinum Neurotoxin A” OR “Neurotoxin A, Botulinum” OR “Botulinum A Toxin” OR “Toxin, Botulinum A” OR “Botulinum Toxin Type A” OR “Botulinum Neurotoxin Type A” OR “Clostridium Botulinum Toxin Type A” OR “Meditoxin” OR “Botox” OR “Neuronox” OR “Oculinum” OR “Vistabex” OR “OnabotulinumtoxinA” OR “Onabotulinumtoxin A” OR “Vistabel” |
| #23 | “Botulinum Toxins” OR “Toxins, Botulinum” OR “Botulinum Neurotoxins” OR “Neurotoxins, Botulinum” OR “Botulinum Toxin” OR “Toxin, Botulinum” OR “Clostridium botulinum Toxins” OR “Toxins, Clostridium botulinum” OR “Botulinum Neurotoxin” OR “Neurotoxin, Botulinum” OR “Botulin” |
| #24 | #22 OR #23 |
| #25 | “Extracorporeal Shockwave Therapy” OR “Extracorporeal Shockwave Therapies” OR “Shockwave Therapies, Extracorporeal” OR “Shockwave Therapy, Extracorporeal” OR “Therapy, Extracorporeal Shockwave” OR “Shock Wave Therapy” OR “Shock Wave Therapies” OR “Therapy, Shock Wave” OR “Extracorporeal Shock Wave Therapy” OR “Extracorporeal High-Intensity Focused Ultrasound Therapy” OR “Extracorporeal High Intensity Focused Ultrasound Therapy” OR “HIFU Therapy” OR “HIFU Therapies” OR “Therapy, HIFU” OR “High-Intensity Focused Ultrasound Therapy” OR “High Intensity Focused Ultrasound Therapy” OR “Shock Wave” OR “ESWT” OR “rESWT” |
| #26 | #16 AND #21 AND #24 AND #25 |

Supplementary table 2 Baseline functional status

| Included studies | **Group** | **MAS** | **FMA** | **MBI** | **VAS** | **PROM** | **BBS** | **SFS** | **Tone** | **Hardness** |
| --- | --- | --- | --- | --- | --- | --- | --- | --- | --- | --- |
| Megna, M.et al. 2019 | T | 3.5±0.2 | - | - | 8.9±0.3 | - | - | - | 29.11±1.43 | 311.65±2.43 |
|  | C | 3.3±0.2 | - | - | 9.2±0.7 | - | - | - | 28.34±1.23 | 312.89±4.25 |
| Santamato, A.et al. 2013 | T | 3.5±0.52 | - | - | 5±1.21 | - | - | 2.37±1.15 | - | - |
|  | C | 3.62±0.5 | - | - | 5.25±1.34 | - | - | 2.56±1.03 | - | - |
| Luyan 2021 | T | 2.52±0.41 | 44.60±7.89 | - | 4.32±1.37 | - | 32.23±3.62 | - | - | - |
|  | C | 2.48±0.37 | 44.52±7.95 | - | 4.28±1.34 | - | 32.20±3.57 | - | - | - |
| Lianghui.et al.2021 | T | 3.28±1.18 | 17.38±1.41 | 40.51±3.53 | - | - | - | - | - | - |
|  | C | 3.21±1.03 | 16.92±2.57 | 41.29±4.32 | - | - | - | - | - | - |
| Duanhaoyang.et al. 2020 | T | 3.27±0.17 | 9.57±4.46 | 26.83±5.69 | - | 25.58±4.52 | - | - | - | - |
|  | C | 3.19±0.84 | 10.36±5.87 | 27.19±6.82 | - | 27.02±4.39 | - | - | - | - |
| Wanghong.et al. 2017 | T | 2.71±0.14 | 13.87±1.36 | 40.36±3.87 | - | - | - | - | - | - |
|  | C1 | 2.63±0.11 | 13.71±1.18 | 41.28±4.38 | - | - | - | - | - | - |
|  | C2 | 2.68±0.13 | 14.06±1.21 | 40.72±3.65 | - | - | - | - | - | - |

Note: T is the treatment group; C is the control group; "-" indicates no report. Muscle tone and hardness were measured by MyotonPro®.

Supplementary table 3 Inclusion and exclusion criteria

| **Included studies** | **Inclusion criteria** | **Exclusion criteria** |
| --- | --- | --- |
| Megna, M.et al. 2019 | Modified Ashworth Scale (MAS)> 2 of the target muscles; no infectious and/or neo-plastic processes; no muscle fibrosis; no focal treatment of spasticity with BTX-A within the last 5 months before recruitment (to be sure end of previous treatment effect). | The presence of fixed contractures, or bony deformities of the affected limbs, previous treatment of spastic muscles with nerve phenolization, previous orthopedic or neurosurgery procedures, and other neurological or orthopedic conditions involving the affected limbs. |
| Santamato, A.et al. 2013 | The inclusion criteria at screening and at the base-line visit were: focal spasticity of finger flexors  measured as ≥2 on the modified Ashworth scale(MAS) , at least a 6-month period from stroke, daily painful muscle spasms measured as≥2 on the spasm frequency scale (SFS), and pain at rest and during limb mobilization measured as ≥3 on the visual analogue scale(VAS) . | Fixed contractures and/or deformities at the wrist and elbow, previous fractures of the paretic upper limb, cognitive impairment, peripheral nervous system disorders/myopathies, pacemaker, pregnant or taking medications that could have an impact on the study or on response to ESWT or ES (e.g., previous BTX-A treatment, GABAergic medications, benzodiazepines, anticoagulants, or muscle relaxants). Patients with structural alterations in the soft tissue (e.g., fibrosis). |
| Luyan 2021 | Meet the criteria of cerebral infarction and cerebral hemorrhage or brain injury in the Academy of Neurology in 2004; aged 20-70 years; confirmed by CT and MRI; modified Ashworth spasm scale (MAS) score≥3, flexor, and tension over 2; patients who can walk 10-15m with the help of helpless walker; patients with stable condition and complete clinical data. | Breast-feeding during pregnancy; infection of injection site; contracture of spastic limbs; patients with peripheral nerve injury, neuromuscular joint transmission disorders, bone and joint diseases; patients with lumbar sacral canal disease and severe osteoporosis; patients with serious organic diseases and malignant tumors; patients with abnormal coagulation mechanism and deep vein thrombosis; infection and skin rupture in the treatment area; patients with cognitive dysfunction, aphasia and mental abnormalities; failure to cooperate with the investigator. |
| Lianghui.et al.2021 | The diagnostic criteria for stroke syndrome, and confirmed as cerebral infarction or cerebral hemorrhage by CT and MRI examination; stable vital signs, good coordination, no motor and sensory aphasia; first onset, disease and onset within 2 weeks; 40-60 years of age; motor function of Brunnstrom on the affected side; muscle spasm on the affected side; conservative treatment over 6 months and poor treatment effect; BTX-A and ESW signed informed consent. | Patients who have been injected with botulinum toxin, or long used antispasmodic drugs such as baclofen or tezanidine; poor cognition and poor family coordination; patients who use aminoglycoside antibiotics or morphine; patients with systemic neuromuscular diseases such as myasthenia gravis, Lambert-Eaton syndrome, and motor neuron disease; patients with pregnancy, severe liver and kidney insufficiency, and severe skin and soft tissue infections. |
| Duanhaoyang.et al. 2020 | Meet the diagnostic criteria for stroke and confirmed; course by imaging examination 2 to 6 months; triceps modified Ashworth scale (modified Ashworth scale, MAS) ≥Grade 2; stable condition can understand and cooperate with informed consent for this study and signed relevant documents. | The ankle on the affected side; ESWT, BTX-A treatment contraindications; unstable vital signs or new lesions causing deterioration; receive other treatment to relieve muscle spasm. |
| Wanghong.et al. 2017 | All meet the diagnostic criteria of "Key Points of Diagnosis of Various Cerebrovascular Diseases" revised by the 4th National Academic Conference of Cerebrovascular Diseases of the Chinese Medical Association in 1995; Patients with cerebral infarction or cerebral hemorrhage confirmed by CT and MRI; Vital signs are stable, Clear awareness, physical examination, no cognitive dysfunction, no serious aphasia; Course of disease: 3-12 months; Between 35 and 65; Motor function of Brunnstrom on the affected limb; Modified Ashworth spasticity in lower limbs, And reach grade 2 or above; Oral anti-spasticity drugs have poor effect or large adverse reactions; Informed consent was signed before botulinum toxin treatment and shock wave treatment. | The disease is unstable, Progressive stroke or secondary stroke; Lower leg local metal holder (except titanium alloy) or install cardiac pacemaker; With severe cardiopulmonary disease and epilepsy; Excluding pregnancy, lactation or previous history of BTX-A injection; With a neuromuscular joint transmission disorder; Patients with severe dysfunction of liver, kidney and other important organs, Patients with severe osteoporosis and malignant tumor patients; Combined with severe cognitive dysfunction, severe aphasia, And even affect the doctor-patient communication; Coagulation dysfunction and deep vein thrombosis; Patients with local infection and skin rupture in the target treatment area. |
